# Supplementary material for: Ni-Catalyzed Enantioselective Intramolecular Mizoroki–Heck Reaction for the Synthesis of Phenanthridinone Derivatives
Source: J Org Chem. 2023 Jun 15;88(13):8203–26. doi: 10.1021/acs.joc.3c00202 (PMC10337041; doi:10.1021/acs.joc.3c00202)
Supplement: Supplementary file 2 — jo3c00202_si_002.zip [file jo3c00202_si_002.zip › Amides (N-MOM)/1i iPr-NMOM-4F-ArBr/1H iPr-NMOM-4F-ArBr 1i/2/pdata/1/email_DR-112-077 F19-22_2_1.pdf]

DR-112-077 F19-22 COSY
COSYGPSW DMSO {D:\nmrusers\malachowski} BM 2

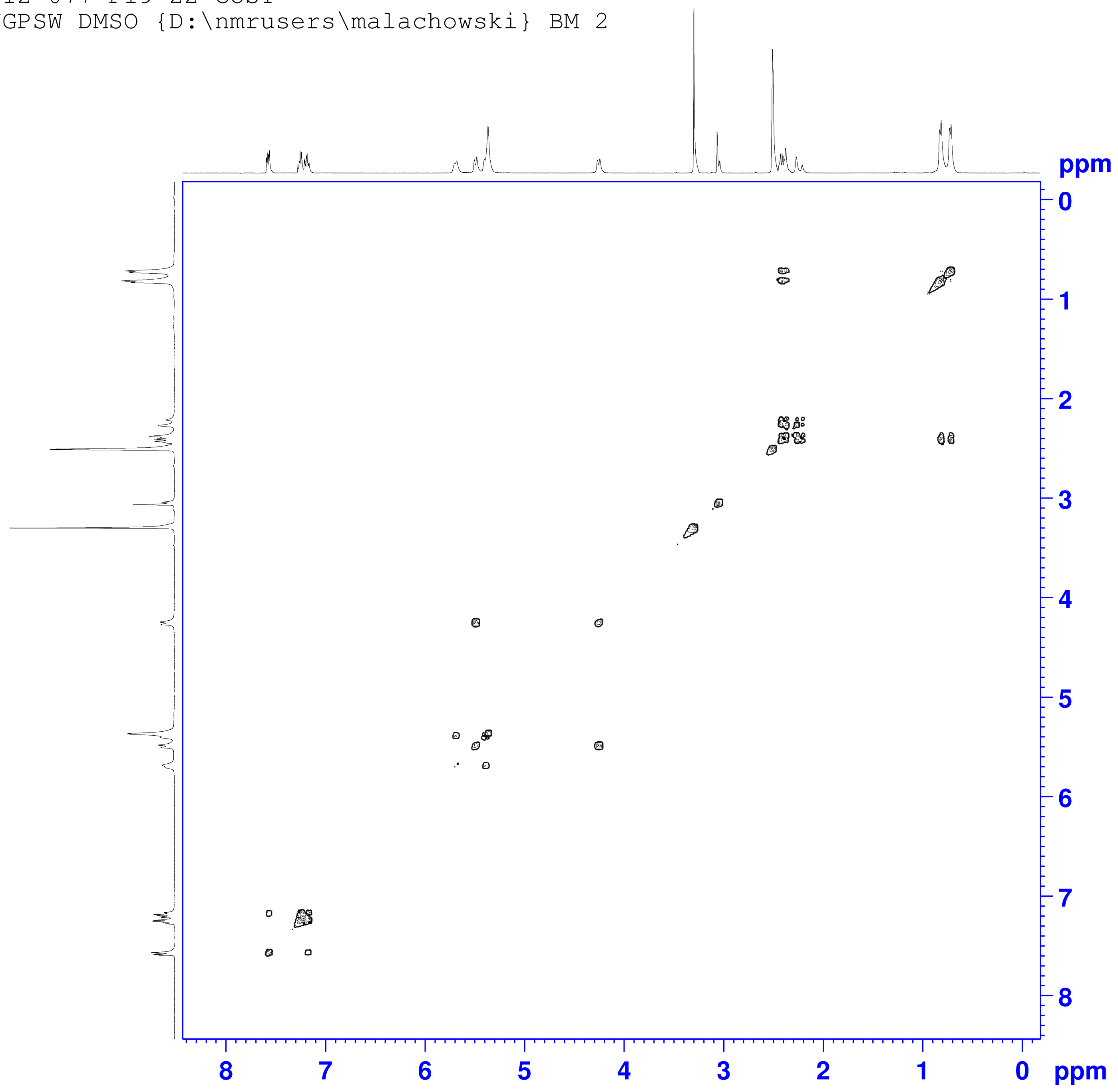

BRUKER

Current Data Parameters
NAME DR-112-077 F19-22
EXPNO 2
PROCNO 1

F2 - Acquisition Parameters
Date_ 20211014
Time 18.25 h
INSTRUM spect
PROBHD Z104450_0352 (
PULPROG cosygpppqf
TD 2048
SOLVENT DMSO
NS 1
DS 16
SWH 3448.276 Hz
FIDRES 3.367457 Hz
AQ 0.2969600 sec
RG 161
DW 145.000 usec
DE 6.50 usec
TE 350.0 K
D0 0.00000300 sec
D1 1.90005803 sec
D11 0.03000000 sec
D12 0.00002000 sec
D13 0.00000400 sec
D16 0.00020000 sec
IN0 0.00029000 sec
TDav 1
SFO1 400.1516515 MHz
NUC1 1H
P0 12.75 usec
P1 12.75 usec
P17 2500.00 usec
PLW1 12.14200020 W
PLW10 2.19309998 W
GPNAM[1] SMSQ10.100
GPZ1 10.00 %
P16 1000.00 usec

F1 - Acquisition parameters
TD 128
SFO1 400.1517 MHz
FIDRES 53.879311 Hz
SW 8.617 ppm
FnMODE QF

F2 - Processing parameters
SI 1024
SF 400.1500000 MHz
WDW QSINE
SSB 0
LB 0 Hz
GB 0
PC 1.40

F1 - Processing parameters
SI 1024
MC2 QF
SF 400.1500000 MHz
WDW QSINE
SSB 0
LB 0 Hz
GB 0
